# Association between vitamin D supplementation and COVID-19 infection and mortality

**DOI:** 10.1038/s41598-022-24053-4

**Published:** 2022-11-12

**Authors:** Jason B. Gibbons, Edward C. Norton, Jeffrey S. McCullough, David O. Meltzer, Jill Lavigne, Virginia C. Fiedler, Robert D. Gibbons

**Affiliations:** 1 Department of Health Policy and Management, Bloomberg School of Public Health, Johns Hopkins University, 615 N. Wolfe Street, Baltimore, MD 21205 USA; 2 Department of Veterans Health Affairs, Center of Excellence for Suicide Prevention, Canandaigua, USA; 3 Department of Economics, University of Michigan, Ann Arbor, USA; 4 National Bureau of Economic Research, Cambridge, USA; 5 Department of Health Management and Policy, University of Michigan, Ann Arbor, USA; 6 Department of Medicine, University of Chicago, Chicago, USA; 7 Wegmans School of Pharmacy, St John Fisher College, Rochester, USA; 8 Department of Dermatology, University of Illinois at Chicago, Chicago, USA; 9 Department of Public Health Sciences (Biostatistics), University of Chicago, Chicago, USA

**Keywords:** Viral infection, Epidemiology

## Abstract

Vitamin D deficiency has long been associated with reduced immune function that can lead to viral infection. Several studies have shown that Vitamin D deficiency is associated with increases the risk of infection with COVID-19. However, it is unknown if treatment with Vitamin D can reduce the associated risk of COVID-19 infection, which is the focus of this study. In the population of US veterans, we show that Vitamin D_2_ and D_3_ fills were associated with reductions in COVID-19 infection of 28% and 20%, respectively [(D_3_ Hazard Ratio (HR) = 0.80, [95% CI 0.77, 0.83]), D_2_ HR = 0.72, [95% CI 0.65, 0.79]]. Mortality within 30-days of COVID-19 infection was similarly 33% lower with Vitamin D_3_ and 25% lower with D_2_ (D_3_ HR = 0.67, [95% CI 0.59, 0.75]; D_2_ HR = 0.75, [95% CI 0.55, 1.04]). We also find that after controlling for vitamin D blood levels, veterans receiving higher dosages of Vitamin D obtained greater benefits from supplementation than veterans receiving lower dosages. Veterans with Vitamin D blood levels between 0 and 19 ng/ml exhibited the largest decrease in COVID-19 infection following supplementation. Black veterans received greater associated COVID-19 risk reductions with supplementation than White veterans. As a safe, widely available, and affordable treatment, Vitamin D may help to reduce the severity of the COVID-19 pandemic.

## Introduction

Vitamin D insufficiency and deficiency affect approximately half of the US population, with increased rates in people with darker skin, reduced sun exposure, people living in higher latitudes in the winter, nursing home residents, and healthcare workers^1^. Populations with low levels of Vitamin D have also experienced higher rates of COVID-19^2–6^.

Despite several studies pointing to an association between low levels of vitamin D and COVID-19^2–7^, limited information is available regarding the potential for supplementation with vitamin D to reduce the risk of COVID-19 infection. Expanding supplementation with vitamin D may present a new and unique opportunity to mitigate global infection rates, given that it is a widely available over-the-counter (OTC), inexpensive, and is associated with relatively few side effects.

We conducted a large-scale pharmacoepidemiologic study of the association between vitamin D_3_ and D_2_ supplementation and the probability of COVID-19 infection and COVID-19 infection ending in mortality within 30-days in the Department of Veterans Administration (VA) in the United States. We also studied whether patient sex, race, vitamin D serum levels, and cumulative D_3_ supplementation dosage modified the association.

## Background

Severe COVID infection is associated with high levels of circulating activated complement fragments^8^ and a prolonged, enhanced IFN gamma producing TH1 response^9^. Excessive complement and IFN gamma are known drivers of tissue injury^10,11^. SARS-CoV2-infected respiratory epithelial cells express and process complement (C)3 to generate C3a and C3b^12^. C3b binds the CD46 receptor on CD4 + T cells to drive TH1 differentiation normally followed by their shutdown. The usual sequence involves the production of IFN gamma alone, then IFN gamma plus IL10, and finally IL10 alone^13^. IL10 production by TH1 cells is critical to the regulation of inflammation^14^. Chauss et al. have recently identified an autocrine/paracrine Vitamin D loop which permits TH1 cells to both activate and respond to Vitamin D as part of the cellular program to shut down IFN gamma and enhance IL10^15^. This finding suggests that the addition of Vitamin D to other immunomodulatory agents may be useful in patients infected with SARS-CoV2.

Several observational studies have shown a strong relationship between infection with COVID-19 and low serum levels of vitamin D^2–7^. An early study of 489 patients with vitamin D lab values in the year before a COVID-19 test found a 77% increased risk of a positive COVID-19 test in those with vitamin D deficiency (25-hydroxycholecalciferol less than 20 ng/mL or 1,25-dihydroxycholecalciferol less than 18 pg/mL)^2^. A separate study found a 59% increased risk of severe COVID-19 infection symptoms among persons with low levels of vitamin D (25-hydroxycholecalciferol less than 30 ng/mL)^3^. Using the same threshold, a third study found a 45% increase in COVID-19 infection and a 95% increase in resulting hospitalizations^4^. A fourth report found a 35% increase in COVID-19 infection rates among patients with vitamin D deficiency (< 20 ng/mL) versus those between 30 and 34 ng/mL and a 53% increase relative to those with values ≥ 55 ng/mL^5^. A fifth large-scale observational study in Israel further confirmed previous findings^6^. More generally, vitamin D has long been known to improve patient immune response. A meta-analysis of 25 randomized controlled clinical trials (RCTs) identified a 12% decrease in acute respiratory tract infections in patients receiving vitamin D supplementation^16^.

Two randomized controlled trials have tested the ability of vitamin D to improve outcomes in patients already infected with COVID-19. First, a small open-label study randomized 76 patients with COVID-19 infections by a 2:1 ratio to treatment with oral calcifediol (a vitamin D_3_ analog) or only standard treatment. Of the 50 patients in the intervention group, only one (2%) required admission to the ICU. In contrast, 26 (50%) of the patients in the standard treatment group required ICU admission^17^. A second randomized controlled trial of treatment with a single oral high dose (200,000 IU) of oral calcifediol in 240 hospitalized patients found no reduction in length of stay^18^. A later reanalysis found a 52% reduction in admissions to intensive care and a 60% reduction in the use of mechanical ventilation among only those intervention group patients with 25(OH)D values ≥ 20 ng/mL^19^.

A recent meta-analysis has shown an association between low serum 25-hydroxy Vitamin D levels and susceptibility to, severity of, and mortality from COVID-19^20^.

Most recently, a retrospective cohort study of all COVID-19 hospitalized patients in Andalusia (n = 15,968) found a 33% reduction in mortality when calcifediol (i.e., Vitamin D_2_) was prescribed 15 days before hospitalization and a 25% reduction in mortality for cholecalciferol (i.e., vitamin D_3_)^21^. Reductions were 27% and 12% when the window was extended to 30 days before hospitalization. A second recent study in Israel (n = 253 patients with pre-infection vitamin D levels) found a 14-fold increase of severe or critical COVID-19 disease in patients with vitamin D deficiency (< 20 ng/ml) relative to patients with normal vitamin D levels (> = 40 ng/ml), adjusted for age, sex, BMI and comorbidities^22^.

Despite the aforementioned findings for an association between vitamin d deficiency and COVID-19 infection, there are other studies that draw the significance of this finding into question in addition to the relationship between supplementation with vitamin D and COVID-19. One meta-analysis of three recent studies found no relationship between vitamin D supplementation and COVID-19 mortality while infected^23^. Another meta-analysis explained that much of the evidence from observational studies that found an inverse association between vitamin D levels and COVID-19 infection risk, severity, and mortality was weak, and that more randomized controlled trials were needed to clarify the relationship^24^. A third meta-analysis was unable to identify a statistically significant relationship between vitamin D supplementation and COVID-19 infection risk, mortality, and ICU admissions^25^.

Given the mixed findings in the literature, there is a need for more research on vitamin D supplementation as prophylaxis or treatment of COVID-19 infection, including potential reductions in mortality. We extend the analysis of this association by analyzing a large cohort of United States military veterans, comparing those prescribed vitamin D_3_ and D_2_ in terms of risk of COVID-19 infection and mortality to matched controls.

## Methods

### Study design

We conducted a retrospective cohort study to determine the association between vitamin D_3_ and D_2_ supplementation and COVID-19 infection and mortality. We estimated the association using a cohort of VA patients who received supplementation with, ergocalciferol (i.e., vitamin D_2_), doxercalciferol (i.e., vitamin D_2_), oral cholecalciferol (i.e., vitamin D_3_), or calcifediol (i.e., vitamin D_3_) for a period before the pandemic (i.e., January 1, 2019–December 31, 2020), and during the pandemic (i.e., March 1, 2020–December 31, 2020), and untreated control patients. Before estimation, we matched treated and control patients one-to-one on their propensity for supplementation separately for vitamin D_3_ and D_2_. We then used Cox proportional hazards models to calculate time-to-COVID-19 infection and mortality within 30-days following infection, conditional on supplementation_._ We also conducted subgroup analyses for D_3_ to determine treatment heterogeneity by race (Black versus white), vitamin D level (0–19 ng/ml, 20–39 ng/ml, and 40 + ng/ml of 25-dihydroxycholecalciferol), and average daily and cumulative supplementation dosage. The smaller size of the D_2_ dataset precluded subgroup analyses.

### Study population

We identified VA patients with at least one VA service or prescription and at least one vitamin D lab test between January 1, 2019, and December 31, 2020, in the Veterans Administration Corporate Data Warehouse (CDW) electronic health records. Among the treated population, patients receiving prescriptions for both vitamin D_3_ and D_2_ during the active period were dropped to reduce spillover between the associations. Further, patients whose first prescription was during the pandemic were dropped from the sample as these patients are unlikely to have been exposed to treatment long enough to obtain significant protection. (Vitamin D levels typically respond to treatment following two months of exposure). This restriction also reduced the potential for patient selection into treatment; patients taking preventative measures, such as initiating supplementation during the pandemic, could be more likely to engage in other preventive behaviors (e.g., mask-wearing) that would confound the association between treatment and COVID-19 infection and mortality. This also eliminated the possibility of immortality bias; infection during the pandemic, but before vitamin D supplementation, could not be counted. After applying all restrictions, we identified 220,265 patients supplemented with vitamin D_3,_ 34,710 supplemented with vitamin D_2_, and 407,860 untreated patients.

We used one-to-one propensity score matching from our restricted patient sample to match vitamin D_3_ treated and vitamin D_2_ treated patients to controls separately. Covariates used to generate propensity scores included the 15 most common indications for vitamin D prescription fills (see Table 1) and patient demographics (i.e., age, race, and gender). Patient race (i.e., Asian, Black, Native American, other race, White) and gender (i.e., male and female) included all available groups reported directly from the Department of Veterans Affairs CDW electronic health records. After matching, we obtained 199,498 vitamin D_3_ treated and matched control pairs and 33,216 vitamin D_2_ treated and matched control pairs.

**Table 1:** Patient sample before and after matching.

Covariate	Vitamin D_2_				Vitamin D_3_			
	Pre-matching		Post-matching restricted sample		Pre-matching		Post-matching restricted sample	
	Control	Treated	Control	Treated	Control	Treated	Control	Treated
N	407,860	34,710	33,216	33,216	407,860	220,265	199,498	199,498
Age	64	58	58	58	64	63	64	63
**Gender**								
Female	8.6%	1.3%	12.4%	12.3%	8.6%	11.0%	9.9%	10.1%
Male	91.5%	98.7%	87.6%	87.7%	91.5%	89.0%	90.1%	89.9%
**Race/ethnicity**								
Asian	1.0%	1.2%	1.2%	1.2%	1.0%	1.7%	1.4%	1.5%
Black	11.8%	36.5%	34.6%	33.9%	11.8%	21.8%	18.1%	17.6%
Native American	1.5%	1.6%	1.6%	1.6%	1.5%	2.0%	1.9%	1.9%
Other race	3.9%	4.0%	4.0%	4.2%	3.9%	4.7%	4.7%	4.6%
White	81.8%	56.7%	58.6%	59.1%	81.8%	69.8%	73.9%	74.4%
**Condition**								
Vitamin D deficient	34.3%	52.8%	50.3%	51.0%	34.3%	50.3%	45.3%	46.2%
Hypertension	66.9%	63.9%	64.2%	63.8%	66.9%	71.1%	71.7%	69.9%
Hyperlipidemia	68.2%	60.3%	60.4%	60.9%	68.2%	70.0%	70.8%	69.5%
Diabetes	27.3%	28.9%	29.2%	28.6%	27.3%	33.8%	33.4%	31.9%
Needs flu vaccine	74.3%	67.0%	66.5%	67.4%	74.3%	76.3%	76.7%	75.7%
Reflux	39.0%	34.6%	34.5%	34.8%	39.0%	42.9%	43.2%	41.7%
Anemia	19.4%	17.2%	17.4%	17.2%	19.4%	22.3%	22.5%	21.2%
Hypothyroidism	14.0%	9.8%	10.1%	10.1%	14.0%	14.0%	14.7%	14.2%
Long-term use of medications	34.8%	28.1%	28.6%	28.5%	34.8%	35.6%	36.4%	35.3%
Limb pain	17.5%	14.7%	14.6%	14.7%	17.5%	19.4%	19.9%	18.7%
Fatigue	21.2%	15.8%	15.8%	16.0%	21.2%	20.3%	21.3%	20.6%
Hypercholesterolemia	18.0%	11.2%	11.2%	11.5%	18.0%	17.2%	18.6%	17.4%
Depression	26.2%	31.7%	30.7%	30.8%	26.2%	36.2%	33.6%	33.0%
Congestive heart failure	8.1%	7.0%	7.2%	7.1%	8.0%	9.0%	9.5%	8.8%
Urinary tract infection	14.0%	12.3%	12.0%	12.4%	14.0%	16.0%	16.3%	15.3%

Treated beneficiaries include all beneficiaries that received vitamin D3 between January 1, 2019 and December 31, 2020 except for beneficiaries that received treatment after infection with COVID-19. These beneficiaries were considered as controls, as were those beneficiaries that never received treatment.

Source: VA and Medicare Claims Data.

In addition to our primary analysis that included all treated and control patients, we also created stratified patient cohorts by gender (male or female), race (Black or white), and vitamin D serum levels (i.e., 0–19 ng/ml, 20–39 ng/ml, and 40 + ng/ml). Black patients typically have greater vitamin D deficiency and COVID-19 infection rates and may benefit from supplementation more than patients of other races^26^. Men have also been found to have lower Vitamin D blood levels than women^27^. Similarly, patients with lower vitamin D serum levels may benefit from supplementation more than patients with higher levels. Further, patients receiving higher dosages are more likely to increase their serum levels to levels necessary for protection against COVID-19 than patients receiving lower dosages^28^. In our vitamin D_3_ sample, we identified 359,081 men and 39,915 women, 71,071 Black patients and 283,248 white patients, and 69,067 patients whose first vitamin D serum lab value was between 0 and 19 ng/ml during the study, 228,093 between 20–39 ng/ml, and 101,836 with 40 + ng/ml. We did not create subgroups for Vitamin D_2_ as the population size was too small to produce reliable estimates.

### Exposure

The primary exposure was vitamin D_3_ or D_2_ supplementation occurring before and after the pandemic began on March 1, 2020. Patients who never received vitamin D_3_ or D_2_ served as the reference group (i.e., the control group). We included all vitamin D_3_ or D_2_ products and dosages in the CDW electronic health records, including combination products containing Vitamin D_3_, such as multivitamins.

To study variation in the association between treatment and COVID-19 infection by vitamin D serum levels, we constructed a categorical variable representing different 25-dihydroxycholecalciferol thresholds (i.e., 0–19 ng/ml, 20–39 ng/ml, and 40 + ng/ml). The blood level used to create the categorical variable was the value from the first 25-hydroxyvitamin D lab test during the study period (i.e., January 1, 2019–December 31, 2020) for each patient (i.e., treated and control).

We created two dosage measures to study the potential for a dose–response relationship. The first measure, cumulative dosage, was constructed by multiplying each prescription dosage by the days supplied. We then aggregated the resulting values across all prescriptions filled by patients during the pandemic (i.e., March 1, 2020–December 31, 2020). The second measure was the average daily dosage, weighted by days supplied during the pandemic. Dosage options included 20 IU, 40 IU, 100 IU, 125 IU, 200 IU, 250 IU, 400 IU, 500 IU, 800 IU, 1000 IU, 2000 IU, 5000 IU, 8000 IU, and 50,000 IU. Given the large dosage values and skewed distributions for both measures, we took the natural logarithm for use in our analysis (see Table S3).

### Outcomes

The primary outcome was a laboratory-confirmed COVID-19 infection as measured by any VA medical record or Medicare claim containing a diagnosis for ICD-10 code U07.1. We also looked at COVID-19 ending in mortality as a secondary outcome. We defined COVID-19 ending in mortality as any death within 30 days following infection. Although death certificate data were unavailable and would be preferable, mortality occurring shortly after infection is likely to be strongly correlated with actual COVID-19 mortality.

### Statistical analysis

We used a Cox proportional hazards model to separately compare vitamin D_3_ and D_2_ supplementation groups to matched controls regarding time to COVID-19 diagnosis and time to COVID-19 diagnosis followed by mortality within 30 days. Estimates from Cox proportional hazards models are in terms of hazard ratios, which are measures of the instantaneous risk of a particular outcome at a given point in time. For the time-to-COVID-19 diagnosis model, we censored all patients on the date of a laboratory-confirmed COVID-19 diagnosis, mortality, or the end of the study period on December 31, 2020. In the time-to-COVID-19 diagnosis followed by mortality within 30-days model we censored patients on mortality or the end of the study period. We then repeated the time-to-COVID-19 diagnosis analysis in our vitamin D_3_ treated and control cohort using stratifications by race (Black versus white), gender (male versus female), vitamin D serum levels (0–19 ng/ml, 20–39 ng/ml, and 40 + ng/ml). In terms of race, we conducted an additional analysis to estimate the race by treatment interaction, with and without adjustment for vitamin D serum level. Our dose–response analyses that used cumulative vitamin D_3_ and average daily dosage were estimated in the entire sample and within the serum-level stratifications. Finally, we conducted a sensitivity analysis that controlled for seasonality as a time-varying covariate.

We conducted all analyses using STATA-17.

This study (VA MIRB # 00701, PI Jill Lavigne) was reviewed and approved under Category 4 exempt determination by the Syracuse VA Medical Center Institutional Review Board in Syracuse, New York, and the VA’s VIREC Office for Medicare data. This research did not meet the criteria for humans subjects research because the VA Corporate Warehouse Data and Medicare claims are de-identified**,** so informed consent was not required. All methods were performed in accordance with the relevant guidelines and regulations.

## Results

Before matching, there were significant differences in the proportion of Black and white VA patients, vitamin D deficient patients, and patients with depression. Following matching, the vitamin D_3_ supplemented and control groups were similar across potential confounders (see Table 1). A larger proportion of Black patients received vitamin D_2_ versus D_3_. In general, patients receiving vitamin D_3_ had more comorbid conditions than those receiving D_2_, see Table 1.

To ensure patients were well matched on observable characteristics likely to predict supplementation, we evaluated covariate balance and common support between supplementation and control patients after matching. Common support was assessed by dividing the propensity score into centiles and comparing treated and control patients before matching. Covariate balance was determined by dividing the sample into quintiles based on the propensity score and comparing treated and control subsamples by each covariate within each quintile. Common support of the propensity score between treated and control subjects and covariate balancing was also achieved (see Tables S1, S2; Figs. S1, S2).

Patient frequencies in each estimation sample, and rates of COVID-19 infection by supplemented versus control patients, are presented in Table 2. In the vitamin D_3_ cohort, the COVID-19 rates were 2.66% for the treated and 3.30% for the controls, while the rates of COVID-19 infection followed by death within 30 days were 0.23% for the treated and 0.35% for the controls. In the vitamin D_2_ cohort, the COVID-19 rates were 2.16% for the treated and 2.97% for the controls, while the rates for COVID-19 infection followed by death within 30 days were 0.20% for the treated and 0.26% for the controls.

**Table 2:** COVID-19 frequencies by patient cohort after 1–1 propensity score matching.

Patient cohort	Patients, N	Total control, N	Treated, N	COVID-19 by control, N (%)	COVID-19 by treated, N (%)
**Vitamin D**_**2**_					
Full patient cohort					
COVID-19	66,432	33,216	33,216	987 (2.97%)	716 (2.16%)
COVID-19 ending in mortality within 30-days	66,432	33,216	33,216	86 (0.26%)	65 (0.20%)
**Vitamin D**_**3**_					
Full patient cohort					
COVID-19	398,996	199,498	199,498	6591 (3.30%)	5315 (2.66%)
COVID-19 ending in mortality within 30-days	398,996	199,498	199,498	689 (0.35%)	462 (0.23%)
Male cohort					
COVID-19	359,081	179,720	179,361	6008 (3.34%)	4858 (2.71%)
Female cohort					
COVID-19	39,915	19,778	20,137	583 (2.95%)	457 (2.27%)
Black cohort					
COVID-19	71,071	36,020	35,051	1295 (3.59%)	900 (2.57%)
White cohort					
COVID-19	283,248	141,229	142,019	4571 (3.24%)	3828 (2.70%)
0–19 vitamin D level cohort					
COVID-19	69,067	29,324	39,743	994 (3.39%)	956 (2.41%)
20–39 vitamin D level cohort					
COVID-19	228,093	112,997	115,096	3621 (3.20%)	3023 (2.63%)
40 + vitamin D level cohort					
COVID-19	101,836	57,177	44,659	1976 (3.46%)	1336 (2.99%)

### COVID-19 infections in the total sample

**Figure 1:**
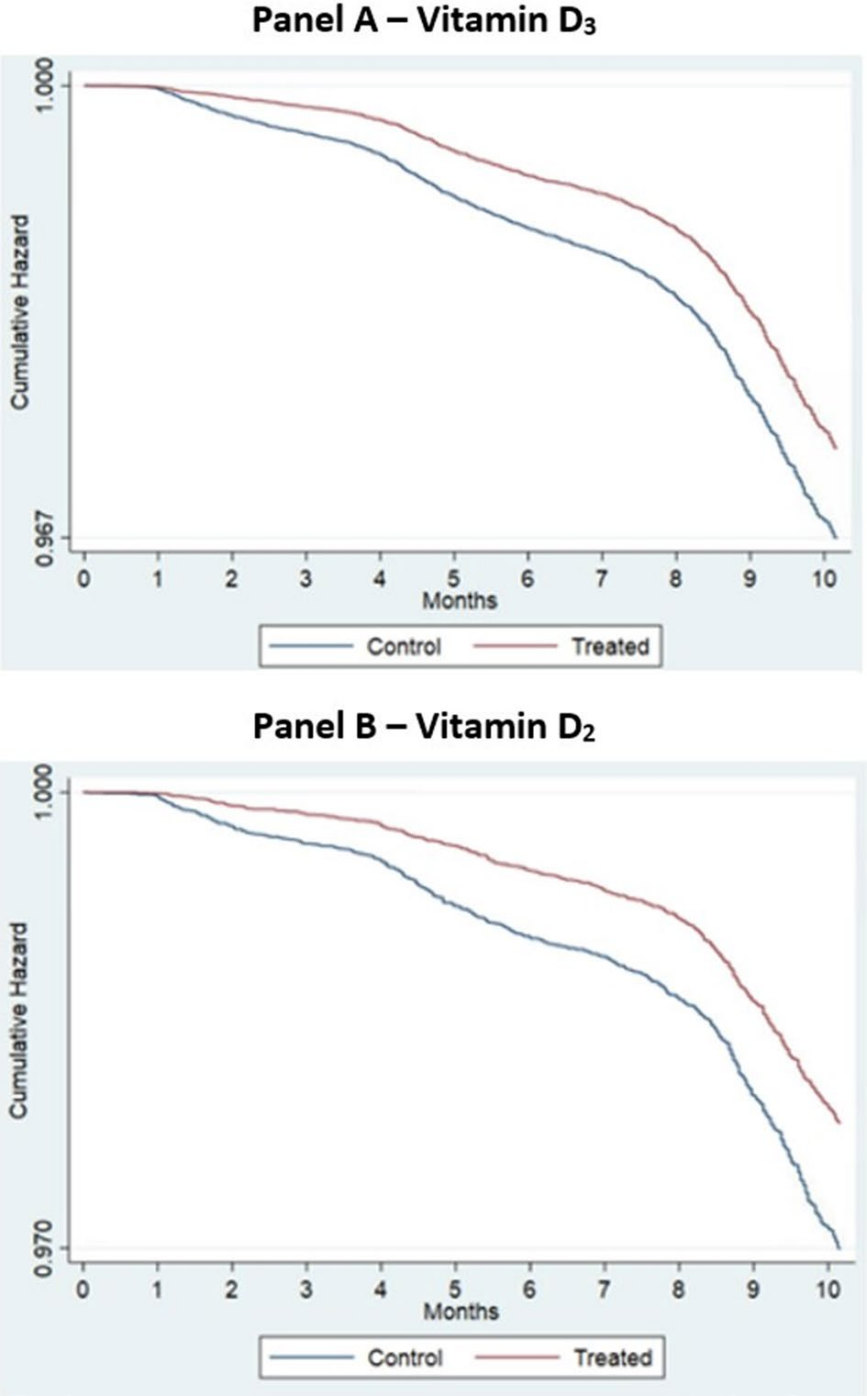
Kaplan Meier curves for vitamin D_3_ and D_2_ supplement patients versus control—time-to COVID-19 infection.

Patients supplemented with vitamin D_3_ and vitamin D_2_ during the pandemic period had an associated 20% and 28% reduction in COVID-19 infection risk relative to untreated controls (D_3_ hazard ratio (HR) = 0.80, [95% CI 0.77, 0.83]; D_2_ HR = 0.72, [95% CI 0.65, 0.79]); see Table 3 and Fig. 1. The resulting vitamin D associations were identical to two decimals places after adjusting for seasonality.

**Table 3:** Hazard ratio estimates.

Patient cohort	Vitamin D_3_ treated versus untreated control (hazard ratio w/ 95% CI)	Vitamin D_2_ treated versus untreated control (hazard ratio w/ 95% CI)
**Full patient cohort**		
COVID-19	0.797*** (0.769, 0.826)	0.720*** (0.654, 0.793)
COVID-19 ending in mortality within 30-days	0.667*** (0.592, 0.750)	0.765 (0.553, 1.057)
**Male cohort**		
COVID-19	0.799*** (0.770, 0.831)	-
**Female Cohort**		
*COVID-19*	0.766*** (0.677, 0.866)	-
**Black cohort**		
COVID-19	0.707*** (0.649, 0.769)	-
**White cohort**		
COVID-19	0.823*** (0.788, 0.859)	-
**0–19 ng/ml vitamin D level cohort**		
COVID-19	0.699*** (0.640, 0.764)	-
**20–39 ng/ml vitamin D level cohort**		
COVID-19	0.811*** (0.772, 0.851)	-
**40 + ng/ml vitamin D level cohort**		
COVID-19	0.856*** (0.798, 0.917)	-
**Cumulative dosage**		
COVID-19	0.981*** (0.978, 0.984)	
**0–19 ng/ml vitamin D level w/cumulative dosage**		
COVID-19	0.970*** (0.963, 0.978)	-
**20–39 ng/ml vitamin D level w/cumulative dosage**		
COVID-19	0.982*** (0.978, 0.986)	-
**40 + ng/ml vitamin D level w/cumulative dosage**		
COVID-19	0.987*** (0.981, 0.993)	-
**Average daily dosage**		
COVID-19	0.969*** (0.965, 0.974)	-
**0–19 ng/ml vitamin D level w/average daily dosage**		
COVID-19	0.953*** (0.942, 0.965)	-
**20–39 ng/ml vitamin D level w/average daily dosage**		
COVID-19	0.971*** (0.965, 0.978)	-
**40 + ng/ml vitamin D level w/average daily dosage**		
COVID-19	0.980*** (0.970, 0.989)	-
**0-19 ng/ml vitamin D level w/ 50,000 IU average daily dosage**		
COVID-19	0.505*** (0.364, 0.701)	-

Parameters expressed, except for cases where cumulative and average dosage are referenced, are for an indicator variable set to 1 if treated and 0 if control. Cumulative dosage is measured as the logarithm of the aggregate dosage during the pandemic period (March 1, 2020–December 31, 2020). Average dosage is measured as the logarithm of the weighted average prescription dosage (weighted by days supplied) during the pandemic period (March 1, 2020–December 31, 2020). Log cumulative dosage and average dosage set to 0 were used for controls. Blood levels were based on the first patient lab value for either treated or control between January 1, 2019 and December 31, 2020).

Source: Veterans Affairs Chronic Data Warehouse Electronic Medical Records data and Medicare Claims Data.

*P < 0.05, **P < 0.01, ***P < 0.001.

The null hypothesis of a proportional hazards model for COVID-19 infection on Vitamin D supplementation was rejected (vitamin D_2_: Chi-Square = 13.98, df = 1, P < 0.001; vitamin D_3_: Chi-Square = 110.95, df = 1, P < 0.001).

### COVID-19 related mortality

**Figure 2:**
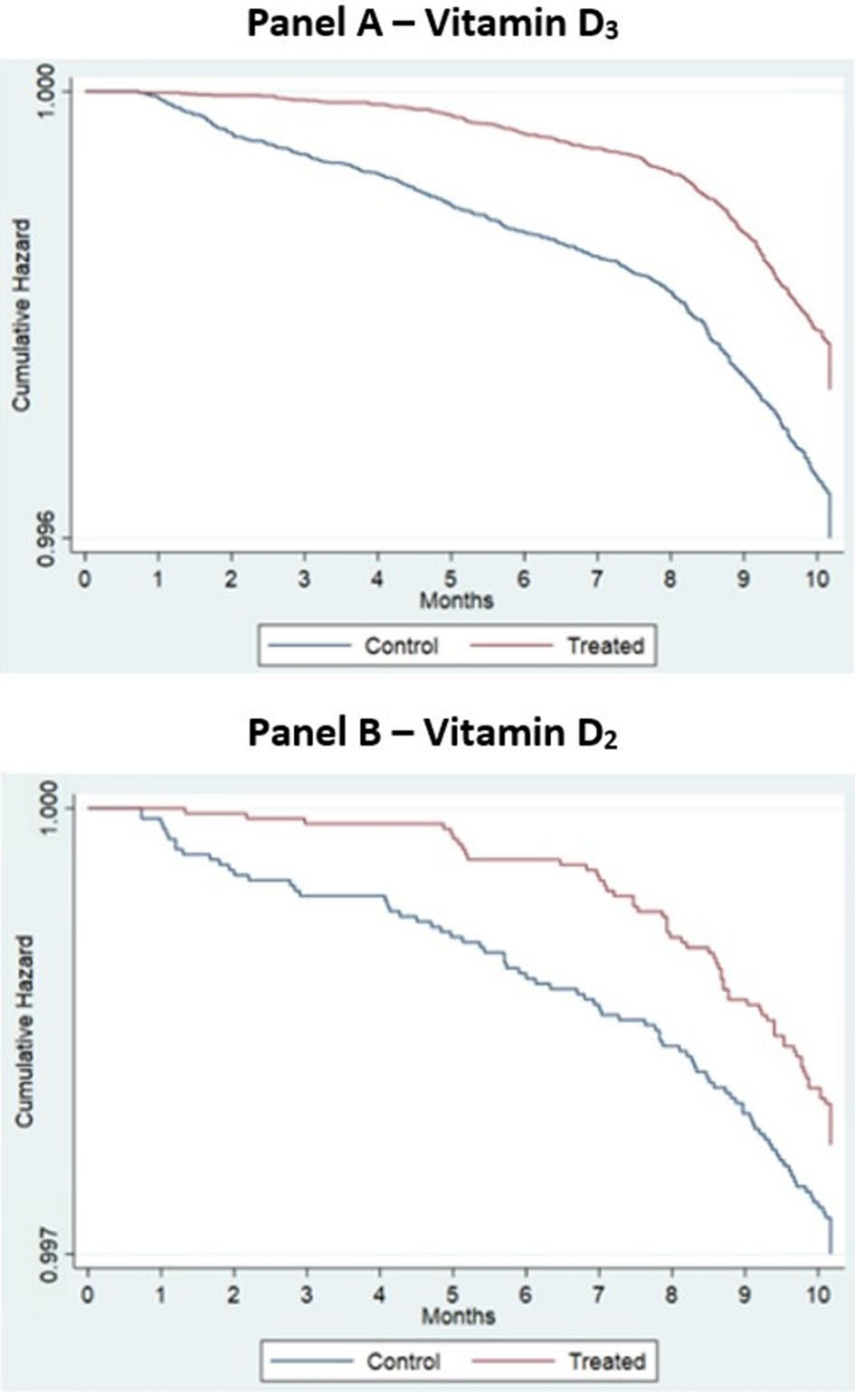
Kaplan Meier curves for vitamin D_3_ and D_2_ supplement patients versus control—time-to COVID-19 infection ending in mortality within 30-days.

Vitamin D_3_ supplementation was associated with a 33% lower risk of COVID-19 infection ending in mortality within 30 days. (HR = 0.67, [95% CI 0.59, 0.75]). However, results for vitamin D_2_ were statistically insignificant (HR = 0.75, [95% CI 0.55, 1.04]); see Table 3 and Fig. 2. The resulting vitamin D associations were identical to two decimals places after adjusting for seasonality.

The null hypothesis of a proportional hazards model for COVID-19 infection ending in death within 30-days on Vitamin D supplementation was rejected (vitamin D_2_: Chi-Square = 10.59, df = 1, P < 0.001; vitamin D_3_: Chi-Square = 69.55, df = 1, P < 0.001).

### Subgroup analyses

#### Male versus female

We found a similar associated reduction in COVID-19 infection rates for male and female patients supplemented with vitamin D_3_ during the pandemic period (male HR = 0.80, [95% CI 0.77, 0.83]; female HR = 0.77, [95% CI 0.68, 0.87]); see Table 3.

#### Black versus white

We found a greater associated reduction in COVID-19 infection rates for Black patients than white supplemented patients relative to controls (Black HR = 0.71, [95% CI 0.65, 0.77]; white HR = 0.82, [95% CI 0.79, 0.86]); see Table 3. The race by treatment interaction was significant (HR = 0.86, [95% CI 0.78, 0.94]). Adjusting for vitamin D serum level did not change the magnitude or significance of the race by treatment interaction (HR = 0.85, [95% CI 0.78, 0.94]).

#### 0–19 ng/ml versus 20–39 ng/ml versus 40 + ng/ml vitamin D serum levels

We found that the associated reduction in COVID-19 infection risk was inversely proportional to vitamin D serum levels (0–19 ng/ml: HR = 0.70, [95% CI 0.64, 0.76] versus 20–39 ng/ml: HR = 0.81, [95% CI 0.77, 0.85] versus 40 + ng/ml: HR = 0.86, [95% CI 0.80, 0.92]); see Table 3. The overall treatment by vitamin D serum level interaction was significant (HR = 1.12, [95% CI 1.06, 1.18]) reflecting a 12% increase in the hazard ratio (smaller treatment related effect) with each increase in serum level category.

#### Cumulative and average daily dosage

Significant dose–response relationships were found for both log_e_ cumulative dosage (HR = 0.981, [95% CI 0.978, 0.984]) and log_e_ average daily dosage (HR = 0.969, [95% CI 0.965, 0.974]). These hazard ratios are for a one natural log unit change; therefore, an approximate tripling (2.718-fold increase) of cumulative dosage is associated with a 2% associated reduction in COVD-19 risk, and a 3% associated reduction for average daily dosage. More intuitively, across the range of cumulative dosages, this represents a 25% associated reduction in COVID-19 risk, and a 27% associated reduction across the range of average daily dosages. The magnitude of the dose–response relation was inversely proportional to vitamin D serum level for cumulative dosage (0–19 ng/ml: HR = 0.970, [95% CI 0.963, 0.978] versus 20–39 ng/ml: HR = 0.982, [95% CI 0.978, 0.986] versus 40 + ng/ml: HR = 0.987, [95% CI 0.981, 0.993]) and average daily dosage (0–19 ng/ml: HR = 0.953, [95% CI 0.942, 0.965] versus 20–39 ng/ml: HR = 0.971, [95% CI 0.965, 0.978] versus 40 + ng/ml: HR = 0.980, [95% CI 0.970, 0.989]) ; see Table 3. Across the dosage range, these statistically significant hazard ratios represent associated reductions of 37%, 24%, and 18% for cumulative dosage, and associated reductions of 38%, 25%, and 18% for average daily dosage. At an average daily dosage of 50,000 IU, there was a 49% associated reduction in COVID-19 infections (HR = 0.51, [95% CI 0.36, 0.70]) in patients with low serum (0–19 ng/ml) vitamin D levels.

## Discussion

Vitamin D supplementation during the pandemic was associated with a significant 20% and 28% reduction in laboratory-confirmed COVID-19 rates for vitamin D_3_ and vitamin D_2,_ respectively. Vitamin D_3_ was associated with a significant 33% decrease in mortality within 30-days of COVID-19 infection. This decrease in COVID-19-related mortality is identical to the 33% observed in the Andalusian data for calcifediol when prescribed 15 days before hospitalization and similar to the 25% associated reduction in mortality for cholecalciferol^21^. For vitamin D_2_ the associated reduction in mortality was 25% but was not statistically significant. These associated reductions in risk are substantial and justify more significant exploration and confirmation using RCTs. This is particularly important given the high rates of vitamin D deficiency in the US population and COVID-19.

There were also striking differences in our results across our patient subgroups. First, compared to white patients, Black patients supplemented with vitamin D_3_ experienced a greater associated reduction in COVID-19 infection rates relative to controls than white patients (29% decrease versus an 18% decrease). Lower serum levels of vitamin D did not explain this finding. Still, these results suggest that expansion of vitamin D supplementation may potentially reduce racial disparities in COVID-19 outcomes. Future research is needed to determine the mechanism by which vitamin D supplementation is more effective among Black patients.

Baseline vitamin D serum levels and cumulative dosage also moderated the effect of vitamin D_3_ treatment. Specifically, patients with lower serum levels receiving higher dosages of vitamin D_3_ experienced the greatest associated reduction in infection. In response to these findings, physicians might consider regularly prescribing vitamin D_3_ to patients with deficient levels to protect them against COVID-19 infection and related mortality. The 50,000 IU dosage may be especially beneficial.

When we extrapolate our results for vitamin D_3_ supplementation to the entire US population in 2020, there would have been approximately 4 million fewer COVID-19 cases and 116,000 deaths avoided. We calculated these values by applying our estimated 20% average reduction in infection and 33% reduction in mortality after infection for vitamin D_3_ to a total of 19,860,000 cases and 351,999 deaths through 2020^29^. In the VA, there have been 343,094 cases and 14,981 known deaths through 10/2/2021. Applying our estimates to the VA, where there would be 69,000 fewer cases and 4900 fewer deaths between March 2020 and October 2021^30^. These back-of-the-envelope calculations may be conservative given possible reductions in COVID-19 transmission due to the general population risk reduction from broader supplementation. Conversely, these estimates may also be inflated if the study population had a higher prevalence of low vitamin D serum levels than the general population due to propensity score matching on supplementation. Still, given our findings, the absence of severe side effects, and the widespread availability of vitamin D_3_ at low cost, vitamin D_3_ presents a unique opportunity to reduce the spread and severity of the COVID-19 pandemic.

## Limitations

Despite successful matching of control patients, there may still be residual confounding. Patients who filled vitamin D_3_ or D_2_ prescriptions may experience better outcomes than others if supplementation is associated with better access to care or the proactive seeking of care, self-care, and behaviors specific to COVID-19 prevention (i.e., social distancing and mask-wearing) than controls. Alternatively, supplemented patients may be more likely to live in colder climates where vitamin D deficiency and COVID-19 rates were elevated in 2020. There are also many important factors we were unable to control for in our statistical analysis that are associated with COVID-19 infection and mortality, including socioeconomic status and weight/obesity. Still, these concerns are lessened by the significant associations between low vitamin D serum levels and higher average and cumulative dosages with improved outcomes.

In addition to residual confounding, our outcomes may suffer from measurement bias. Many cases of COVID-19, especially in the early pandemic period, were not diagnosed due to a lack of available testing. Therefore, there are some infected veterans that received care for their symptoms in the VA or Medicare that may not have been diagnosed. Similarly, positive cases identified by non-VA and non-Medicare providers may be unobserved. Our association would be overstated if vitamin D-supplemented patients were disproportionately underdiagnosed than control patients. This is because patients with undetected infections would be unlikely to be reinfected when testing availability expanded later during our study period. Second, our measure of COVID-19 ending in death within 30-days could also be inaccurate because we did not have access to death certificate data to confirm the actual cause of death. However, many deaths immediately following COVID-19 infection are likely to be at least partially related.

The availability of vitamin D_3_ without a prescription also limits our ability to ensure we have fully categorized dosage and supplementation intensity. We may have misclassified patients that received vitamin D_3_ over the counter as controls. On the contrary, supplement prescriptions may be filled by veterans that do not end up taking them, making some treated patients more similar to controls. In these respects, our estimates would represent lower bounds on the actual impact of vitamin D_3_.

We note that the proportional hazards tests for both the COVID-19 infection risk model and the infection risk ending in death within 30-days were significant, indicating a lack of proportionality of the Vitamin D supplementation association. However, an inspection of the Kaplan–Meier curves in Figs. 1 and 2 reveals little to no difference between supplemented and control veterans within the first month of treatment (i.e., March 2020 when the pandemic began), which steadily increases within three months of follow-up and is relatively constant thereafter. These differences explain the non-proportionality of the Vitamin D supplementation association.

Our findings may also not generalize to new variants, such as the COVID-19 delta variant that became dominant by mid-2021. The delta variant spreads faster and is more deadly than variants that existed during our study period, which may weaken the associations we observed. Further research with updated data will be needed to establish the continued relevance of our findings to new variants, including omnicron. However, given the ability of vaccines to prevent infection with the delta-variant and biologic similarities between the delta-variant with previous strains, we are hopeful that our results will extend at least in part to newer variants. Still, the period used in our study has the advantage of preceding the general availability of vaccines and, therefore, may provide better estimates of the association between vitamin D supplementation and COVID-19 infection and mortality.

Finally, our results are associations. RCTs are ultimately needed to establish a causal link between vitamin D_3_ and D_2_ supplementation and COVID-19 infection and death. Following RCT results, our findings will help confer the generalizability of RCT results to large populations of real-world patients.

## Conclusions

Among VA patients, vitamin D_3_ and vitamin D_2_ supplementation reduced the associated risk of COVID-19 infection by 20% and 28%, and COVID-19 infection ending in death within 30-days by 33% and 25%. Black veterans receiving supplementation had a larger associated reduction than whites, although both were statistically significant, and the difference was not accounted for by differences in vitamin D serum levels. Patients with low vitamin D levels at baseline benefited more from supplementation than patients with higher serum levels. Finally, patients receiving higher cumulative dosages and higher average daily dosages had a greater associated reduction in COVID-19 infection rates than patients receiving lower dosages conditional on similar vitamin D serum levels. The most substantial dose–response relation was found in patients with the lowest vitamin D serum levels. As a widely available, inexpensive, and safe treatment, vitamin D_3_ could be a helpful tool for reducing the spread of COVID-19 infection and related mortality and reducing racial disparities in COVID-19 outcomes. Our findings are especially relevant to the US population, given that about half of Americans are estimated to have sub-optimal vitamin D serum levels.

## Supplementary Information

Supplementary Information.

## Data Availability

The data that support the findings of this study are available from the United States Department of Veterans Affairs, but restrictions apply to the availability of these data, which were used for the current study, and so are not publicly available. Data are however available from the authors upon reasonable request and with permission of the United States Department of Veterans Affairs.
