# Supplementary material for: Association between vitamin D supplementation and COVID-19 infection and mortality
Source: Sci Rep. 2022 Nov 12;12:19397. doi: 10.1038/s41598-022-24053-4 (PMC9653496; doi:10.1038/s41598-022-24053-4)
Supplement: Supplementary file 1 — Supplementary Information. [file 41598_2022_24053_MOESM1_ESM.docx]

**Supplementary Appendix**

**Figure S1. Evaluation of Common Support for Treated and Control Patients by Centile of Propensity Score – Vitamin D_2_**


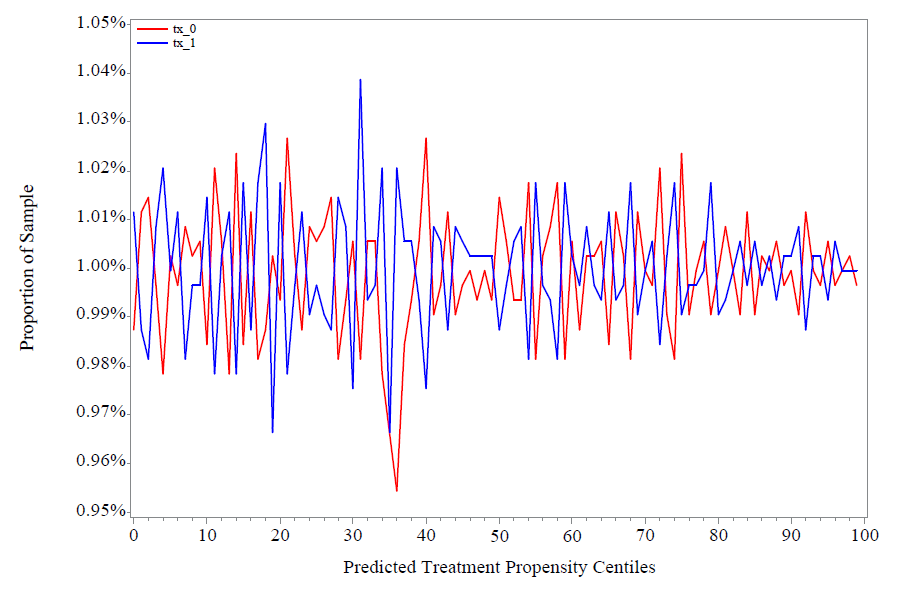


**Notes**: tx_1 represents Vitamin D_3_ treated patients between January 1, 2019 and December 31, 2020. Tx_0 represents untreated controls over the same period. Common Support was evaluated to ensure strong matches were obtained following 1-1 patient propensity score matching on predicted Vitamin D_3_ treatment probabilities.

**Sources**: VA EHR and Medicare Claims Data.

**Figure S2. Evaluation of Common Support for Treated and Control Patients by Centile of Propensity Score – Vitamin D_3_**


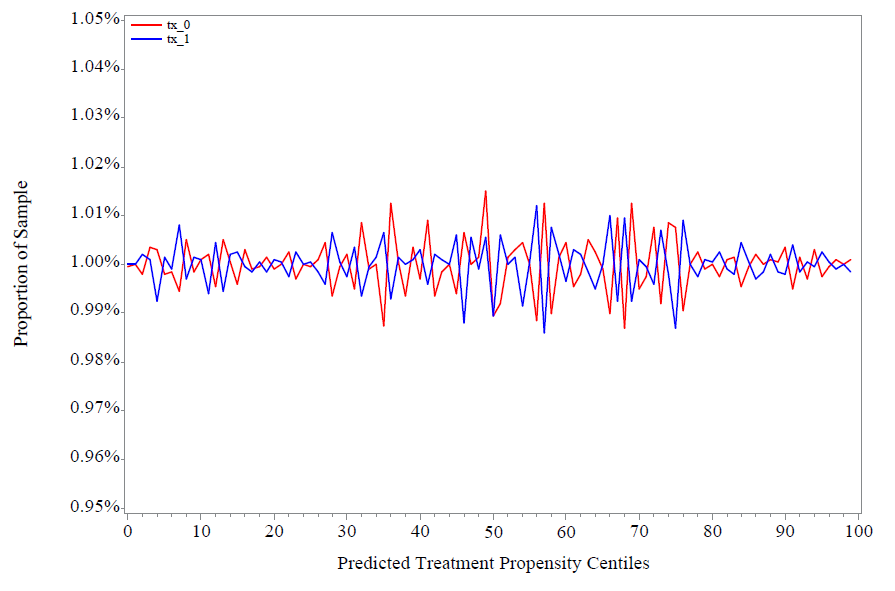


**Notes**: tx_1 represents Vitamin D_3_ treated patients between January 1, 2019 and December 31, 2020. Tx_0 represents untreated controls over the same period. Common Support was evaluated to ensure strong matches were obtained following 1-1 patient propensity score matching on predicted Vitamin D_3_ treatment probabilities.

**Sources**: VA HER and Medicare Claims Data.

**Table S1. Evaluation of Balance in Covariates used in the 1-1 Propensity-Score Matching Model After Matching – Vitamin D_2_**

**
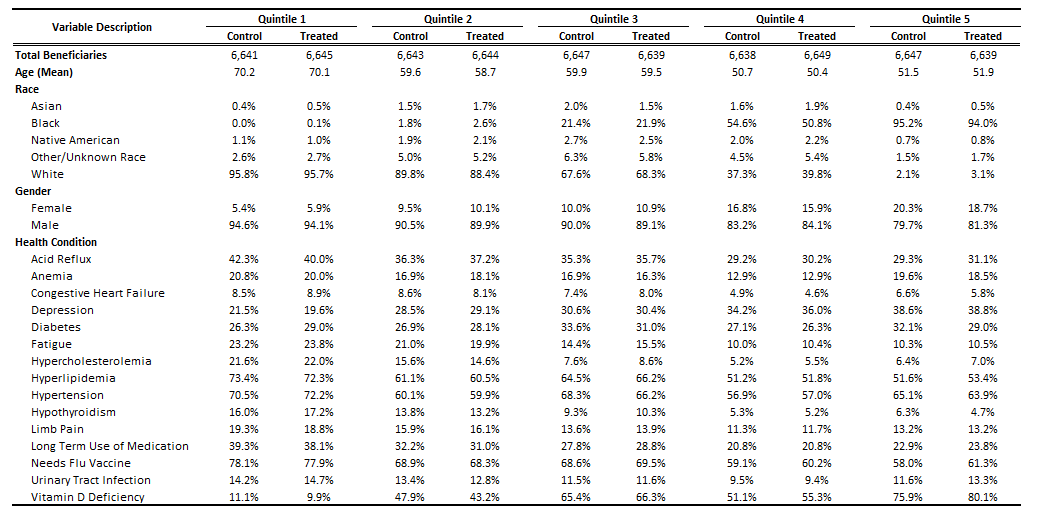
**

| **Notes**: Each cell represents average values of covariates in each quintile of Vitamin D_3_ treatment propensity among treated and control patients. Covariate balance was evaluated to determine minimal patient characteristic differences following 1-1 propensity score matching on patient Vitamin D_3_ treatment probabilities.  **Sources**: VA and Medicare Claims Data |
| --- |

**Table S2. Evaluation of Balance in Covariates used in the 1-1 Propensity-Score Matching Model After Matching – Vitamin D_3_**

**
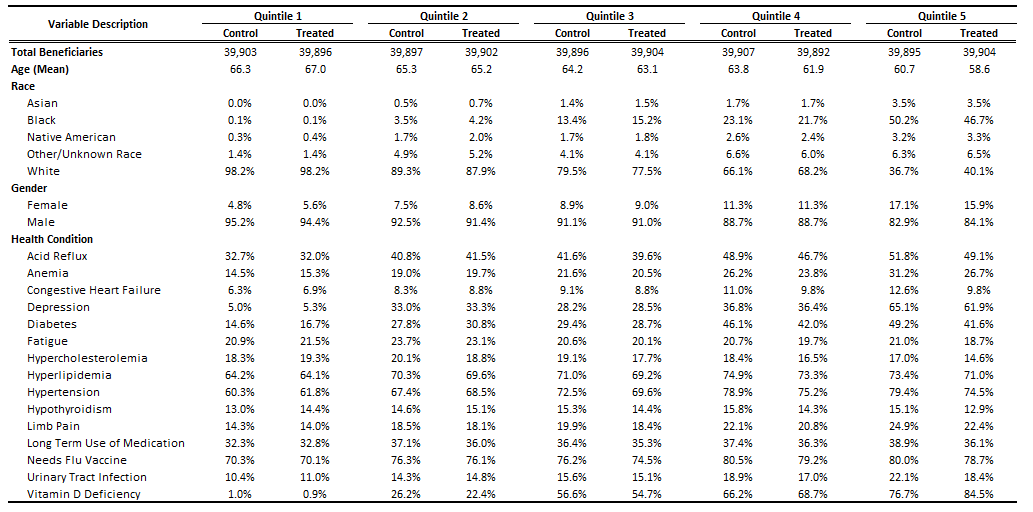
**

| **Notes**: Each cell represents average values of covariates in each quintile of Vitamin D_3_ treatment propensity among treated and control patients. Covariate balance was evaluated to determine minimal patient characteristic differences following 1-1 propensity score matching on patient Vitamin D_3_ treatment probabilities.  **Sources**: VA and Medicare Claims Data |
| --- |

**Table S3. Average and Standard Deviations of Vitamin D Average Dosage and Cumulative Dosage Variables**

| **Dosage Type** | **Vitamin D_2_** | **Vitamin D_3_** |
| --- | --- | --- |
| **Average Dosage** | | |
| Mean | 49,979 | 2290 |
| Standard Deviation | 985 | 6848 |
| **Cumulative Dosage** | | |
| Mean | 632,210 | 151,330 |
| Standard Deviation | 296,314 | 246,531 |

**Sources**: VA and Medicare Claims Data
